# The Significance of miRNA-375 in β-Cell Function and Autoimmune Destruction in Type 1 Diabetes Mellitus: A Review Article

**DOI:** 10.2174/0115748871424258251202164213

**Published:** 2026-02-27

**Authors:** Sariya Khan, Hader I Sakr, Tarek Atia, Redwan Fahad Saaty, Husna Irfan Thalib, Tasneem Mohammed Alsayed, Jakleen Z. Abujamai, Mustafa E Motawee

**Affiliations:** 1 General Medicine Practice Program, Batterjee Medical College, Jeddah, 21442, Saudi Arabia;; 2 Department of Medical Physiology, Faculty of Medicine, Cairo University, Cairo, Egypt;; 3 Department of Medical Physiology, General Medicine Practice Program, Batterjee Medical College, Jeddah, 21442, Saudi Arabia;; 4 Department of Medical Laboratory Sciences, College of Applied Medical Sciences, Prince Sattam Bin Abdulaziz University in Al‐Kharj, Saudi Arabia;; 5 Department of Histology, General Medicine Practice Program, Batterjee Medical College, Jeddah, 21442, Saudi Arabia;

**Keywords:** Type 1 Diabetes Mellitus, Autoimmune Disease, Pancreatic ?-Cells, miRNA-37

## Abstract

**Background:**

Type 1 diabetes mellitus (T1DM) results from progressive damage of pancreatic β-cells, with insufficient insulin production and an inability to regulate glucose levels. The autoimmune destruction of β-cells reduces their functional mass, resulting in chronic hyperglycemia and its associated complications. Recently, microRNAs (miRNAs) are considered significant modulators of gene expression and have been identified in various cellular processes and pathways. Aim: This review article aims to provide a comprehensive explanation of how miRNA-375 influences β-cell biology and explores its potential as a therapeutic target in T1DM. By detailing the intricate interplay between miRNA-375, β-cell function, and autoimmunity, this article aids in developing new guidelines for the early detection, prevention, and possible treatment of T1DM.

**Materials and Methods:**

Electronic databases including PubMed/Midline, Scopus, and Google Scholar were searched for case reports and series, case control, cohort, cross-sectional studies, and reviews from the database's inception to December 2024.

**Results:**

The contribution of miRNAs in the onset and progression of T1DM has garnered considerable attention in recent years. Among these miRNAs, miRNA-375 is particularly significant due to its effect on β-cell actions and apoptosis. Changes in miRNA-375 levels were noted during both the preclinical and clinical phases of T1DM.

**Conclusion:**

Developing reliable and non-invasive methods for monitoring miRNA-375 levels is critical for translating research findings into practical clinical tools. Future studies should focus on creating highly sensitive and specific assays for measuring miRNA-375.

## 1 INTRODUCTION

Pancreatic β-cells are responsible for maintaining glucose homeostasis through the production and release of insulin, thereby regulating energy metabolism and preventing hyperglycemia [1, 2]. The delicate balance maintained by β-cells is essential for overall metabolic health and for preventing various metabolic disorders [1-3]. Type 1 diabetes mellitus (T1DM) is a multifactorial condition resulting from the gradual loss of pancreatic β-cells, typically occurring over several years, leading to insufficient insulin production and impaired glucose regulation [4-6].

Recently, microRNAs (miRNAs) have emerged as significant regulators of gene expression and are implicated in numerous cellular processes and pathways. These small, non-protein-coding RNA molecules interact with specific messenger RNA (mRNA) targets to initiate mRNA degradation and inhibit translation. In T1DM, miRNAs have been identified at various stages of disease pathogenesis, including β-cell function, immune responses, and overall disease progression. Research into miRNAs in T1DM has garnered increasing interest, as they are potential biomarkers for early disease detection and targets for innovative therapeutic strategies. Several miRNAs are differentially expressed in T1DM compared to healthy individuals, indicating their involvement in disease processes. Their presence in biological fluids makes them promising non-invasive diagnostic candidates for prognosis [7].

Among these, microRNA-375 (miR-375) has attracted particular attention, not only in diabetes but also in metabolic and inflammatory disorders. miR-375 regulates insulin secretion, β-cell growth, and survival, and is dysregulated under conditions, such as glucotoxicity, obesity, and metabolic stress. For example, alterations in circulating miR-375 have been associated with improved insulin sensitivity and glucose metabolism in diet-induced weight loss studies, highlighting its potential as a biomarker of metabolic stress and disease risk [8, 9].

Within β-cell biology, miR-375 is recognized as a critical regulator of β-cell function [10-12]. It plays key roles in β-cell development, proliferation, insulin biosynthesis, glucose-stimulated insulin secretion, and the maintenance of β-cell mass [11, 12]. Moreover, miR-375 influences the expression of genes involved in apoptosis and cellular stress, which may contribute to β-cell destruction in T1DM [10, 13, 14]. Understanding the relationship between miR-375 and β-cell function is therefore essential for developing strategies to prevent or delay β-cell loss.

Several approaches are under investigation to modulate miR-375 expression or activity, including miRNA mimics, antagomirs, and small molecule inhibitors. These strategies aim to enhance the protective effects of miR-375 on β-cells or mitigate its detrimental impact in the context of autoimmune β-cell destruction [11, 14]. Beyond its therapeutic potential, miR-375 may serve as a biomarker for T1DM progression and treatment response. Circulating levels of miR-375, in the bloodstream or pancreatic tissue, may reflect β-cell function and indicate the efficacy of interventions designed to preserve β-cell mass [12, 14, 15].

This review aims to provide a comprehensive overview of how miR-375 influences β-cell biology, function, and autoimmunity. By elucidating its role, the goal is to inform strategies for early detection, prevention, and potential treatment of T1DM, ultimately preserving β-cell mass, enhancing insulin secretion, and improving the quality of life for patients affected by this debilitating autoimmune disease.

## 2 MATERIALS AND METHODS

Electronic databases, including PubMed/Medline, Scopus, and Google Scholar, were searched for case reports and series, case-control, cohort, and cross-sectional studies, as well as reviews, from the inception of the databases to December 2024. Studies were included if they were written in English and relevant to the objectives of this review, with no restrictions on publication date, population category, or detection method. The search terms used were “miRNA-375,” “T1DM,” “β-cell dysfunction,” and “autoimmune destruction.”

## 3 ROLE OF MIRNA-375 IN β-CELL FUNCTION

**Fig. (1):**
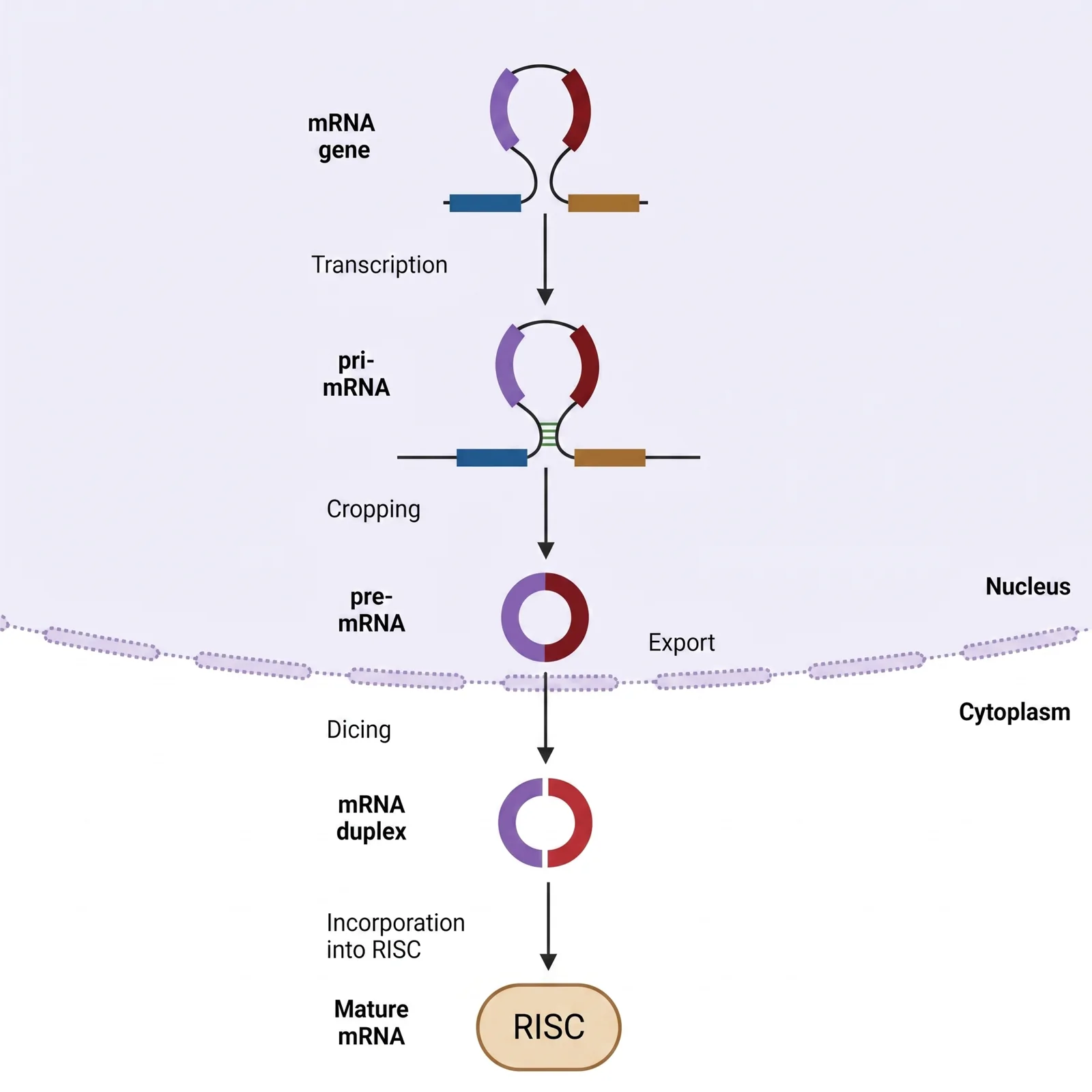
Overview of the biogenesis of miRNA-375 [17].

miRNA-375 is transcribed as a primary miRNA by RNA polymerase II and is subsequently processed in the nucleus by the Drosha-DGCR8 complex to generate a precursor miRNA (pre-miRNA) (Fig. **1**). The pre-miRNA is then exported to the cytoplasm by Exportin-5, where it undergoes further processing by Dicer to produce the mature miRNA. The mature miRNA is incorporated into the RNA-induced silencing complex (RISC), enabling it to bind to the 3′-untranslated regions (3′-UTRs) of target mRNAs through sequence complementarity [16-18]. Depending on the degree of complementarity with the target mRNA, miRNA-375 mediates either translational repression or mRNA degradation. Through this mechanism, miRNA-375 regulates the expression of multiple genes critical for β-cell function. Its role in regulating insulin secretion occurs *via* several mechanisms. Studies using miR-375 knockout (KO) mice have shown impaired glucose-stimulated insulin secretion, highlighting its essential role in β-cell exocytosis [12, 19].

miRNA-375 targets phosphoinositide-dependent protein kinase-1 (Pdk1), a key component of the insulin signaling pathway that is critical for cell growth and survival [11, 12, 20]. By repressing Pdk1, miRNA-375 modulates β-cell proliferation, ensuring an optimal population size to meet physiological demands [11, 12, 20-22]. Additionally, miRNA-375 regulates the expression of the transcriptional coactivator Yes-associated protein 1 (YAP1), which promotes β-cell proliferation [23, 24]. miRNA-375 primarily suppresses YAP1 to prevent excessive β-cell proliferation and maintain controlled growth, a regulation essential for preserving β-cell functionality and preventing tumorigenesis [24]. Furthermore, miRNA-375 modulates pathways that balance pro-apoptotic and anti-apoptotic signals by regulating target genes, thereby supporting β-cell survival under both physiological and stressful conditions [11, 25, 26].

β-cells are particularly susceptible to metabolic and oxidative stress due to their high metabolic activity. miRNA-375 contributes to β-cell resilience under these stressful conditions, enhancing survival in unfavorable environments. Dysregulation of miRNA-375 has been linked to increased β-cell vulnerability to apoptosis, particularly in the context of diabetes mellitus [10, 11, 27-29]. For example, during chronic hyperglycemia in type 2 diabetes (T2DM), β-cells experience elevated oxidative stress, which can increase apoptosis and reduce insulin secretion [24]. miRNA-375 targets and suppresses pro-apoptotic genes, such as Bcl-2-interacting mediator of cell death (Bim), thereby promoting cell survival under stress. It also alleviates endoplasmic reticulum stress by modulating unfolded protein response pathways, which are critical for maintaining insulin biosynthesis and secretion under high-glucose conditions [8, 22-26].

However, the protective role of miRNA-375 has limitations. Under severe or prolonged metabolic stress, including uncontrolled diabetes or chronic inflammation, miRNA-375 expression becomes dysregulated. Inflammation associated with metabolic disorders, particularly obesity-induced inflammation, can exacerbate this dysregulation. Elevated pro-inflammatory cytokines, such as tumor necrosis factor-alpha (TNF-α) and interleukin-1 beta (IL-1β), are linked to altered miRNA-375 levels. While these changes may initially aid stress adaptation, prolonged dysregulation ultimately contributes to β-cell dysfunction. This dual role of miRNA-375, supporting β-cell survival under moderate stress but failing under extreme or chronic stress, underscores its critical involvement in the progression of diabetes [8, 27, 30].

## 4 ROLE OF MIRNA-375 IN AUTOIMMUNE DESTRUCTION OF Β-CELLS

### 4.1 miRNA-375 and Immune Cell Regulation

Immune cell infiltration into pancreatic islets is a hallmark of T1DM. The recruitment of immune cells, particularly T cells, macrophages, and dendritic cells, is driven by the production of pro-inflammatory cytokines and chemokines [31, 32]. Recent studies suggest that miRNA-375 plays a role in regulating immune cell trafficking and infiltration during T1DM development [33].

Macrophages are critical in initiating and resolving inflammation in autoimmune diseases. miRNA-375 modulates macrophage polarization, influencing the balance between pro-inflammatory M1 and anti-inflammatory M2 phenotypes. In T1DM, a shift toward the M1 phenotype is associated with increased tissue damage and immune cell infiltration in the pancreas [34, 35]. miRNA-375 inhibits M1 polarization by targeting signal transducer and activator of transcription 3 (STAT3), a key regulator of M1 differentiation [36-38]. This inhibition reduces the production of pro-inflammatory cytokines, such as TNF-α, potentially limiting immune cell infiltration into pancreatic islets and mitigating tissue damage [39, 40].

T cells, particularly autoreactive CD4+ T cells, play a central role in T1DM pathogenesis [31, 39]. miRNA-375 regulates T cell differentiation and effector functions [37, 39]. Nag *et al*. [40] reported that miRNA-375 inhibits the expression of Foxp3, a transcription factor essential for regulatory T cell (Treg) development. This inhibition promotes the differentiation of pro-inflammatory Th1 and Th17 cells, which secrete cytokines, such as interferon-gamma (IFN-γ) and IL-17. These cytokines recruit additional immune cells to pancreatic islets, exacerbating β-cell destruction [31, 40].

Furthermore, miRNA-375 downregulates TNF-α and IL-17 expression in autoimmune disease models. By targeting key components of the nuclear factor kappa B (NF-κB) signaling pathway, miRNA-375 inhibits the production of these pro-inflammatory cytokines, which are critical for immune cell activation and β-cell apoptosis in T1DM [41, 42]. Chen *et al*. [43] also demonstrated that miRNA-375 suppresses IL-6 secretion in macrophages, further supporting its role in regulating inflammation and immune cell infiltration.

### 4.2 miRNA-375 as a Mediator of Β-cell Apoptosis in T1DM

The development of T1DM is characterized by the destruction of pancreatic β-cells, a process primarily driven by immune cell infiltration and the release of inflammatory cytokines that trigger β-cell apoptosis [31, 32, 41]. miRNA-375 has been implicated in the modulation of both pro-apoptotic and anti-apoptotic pathways, thereby influencing the fate of β-cells [11]. When miRNA-375 is overexpressed, it promotes β-cells apoptosis by targeting key proteins involved in cell survival. One such target is Pdk1, which plays a crucial role in insulin signaling. The inhibition of *Pdk1* by miRNA-375 results in the phosphorylation of PKB and GSK3α/β, ultimately impairing glucose-induced insulin secretion [44, 45]. Conversely, when miRNA-375 is deficient, β-cells exhibit increased resistance to glucolipotoxicity-induced cell death, suggesting that the absence of miRNA-375 may protect β-cells from apoptosis under specific stress conditions. Furthermore, miRNA-375 regulates essential transcription factors involved in β-cell differentiation, including *Hnf1βb*, *Pax6*, *Insm1*, Gata*6*, *Pdpk1*, and *Insr*, indicating its role in both β-cell survival and development [46]. Therefore, miRNA-375 has a multifaceted role in regulating β-cell survival in T1DM, influencing both apoptosis and β-cell differentiation through various pro-apoptotic and anti-apoptotic pathways.

### 4.3 Changes in Circulating miRNA-375 Levels in T1DM Progression

The role of miRNAs in the onset and progression of T1DM has garnered considerable attention in recent years. Among these miRNAs, miRNA-375 is particularly significant due to its involvement in regulating β-cell apoptosis and function. Changes in miRNA-375 levels have been observed during both the preclinical and clinical phases of T1DM, indicating that this miRNA may play a crucial role in the disease's development [45]. In the early preclinical stages of T1DM (before the onset of clinical symptoms), the immune system begins its attack on pancreatic β-cells. Research utilizing animal models, particularly the non-obese diabetic (NOD) mouse model, has revealed that miRNA-375 levels are altered during this initial phase of β-cell destruction. In NOD mice, miRNA-375 is upregulated in the serum, suggesting its potential involvement in early immune activation and β-cell injury [47]. The increased levels of miRNA-375 during this stage indicate that it could serve as an early biomarker for the onset of T1DM [47-51]. Studies on KO diabetic mice have further confirmed these findings, demonstrating that miRNA-375 levels change in response to β-cell damage. These alterations in miRNA-375 levels may reflect the ongoing immune-mediated destruction of β-cells in the preclinical phase, preceding the onset of hyperglycemia. Similarly, in human studies, serum miRNA-375 levels have been measured in children diagnosed with early-onset T1DM who had already tested positive for autoantibodies indicative of β-cell damage. Interestingly, while miRNA-375 levels were elevated, they did not correlate with other common diabetes biomarkers at this stage, suggesting that miRNA-375 may provide unique insights into the early phases of the disease [48, 50]. Fig. (**2**) illustrates the changes in circulating miRNA-375 levels during the progression of T1DM.

In addition to its role in immune-mediated β-cell destruction, miRNA-375 is also influenced by fluctuations in glucose levels. Studies involving human islets exposed to high glucose concentrations have demonstrated that miRNA-375 is downregulated under chronic hyperglycemic conditions, commonly referred to as glucotoxicity. This observation suggests that miRNA-375 may be a component of the cellular stress response triggered by elevated glucose levels. Conversely, in controlled models of T1DM, miRNA-375 is upregulated, further supporting its potential role in the pathophysiology of the disease. These findings highlight the intricate regulation of miRNA-375 in β-cells and indicate that glucotoxicity may affect its expression [9, 46]. In patients with T1DM, circulating levels of miRNA-375 are significantly elevated compared to healthy controls. Research has shown that these elevated levels are present during the early stages of the disease and correlate with increased concentrations of pro-inflammatory cytokines, such as TNF-α and IFN-γ, which are involved in the autoimmune destruction of β-cells. The relationship between miRNA-375 levels and inflammatory markers suggests that miRNA-375 could serve as a valuable biomarker for monitoring disease progression and assessing the severity of β-cell loss in T1DM [12, 47]. Changes in miRNA-375 levels during both the preclinical and clinical stages of T1DM suggest that it may serve as an early biomarker of disease onset and progression. Its upregulation in the presence of inflammatory cytokines further underscores its potential as a therapeutic target. However, further research is necessary to fully understand the complex regulation and broader implications of miRNA-375 in T1DM [52].

## 5 MOLECULAR TARGETS OF MIRNA-375

Experimental data support the complex role of miRNA-375 in β-cell function, survival, and insulin secretion, highlighting its involvement in both direct targets and broader regulatory networks. miRNA-375 represses myotrophin, a gene that plays a crucial role in the exocytosis of insulin granules [10, 53]. Functional studies have demonstrated that elevated levels of Mtpn impair vesicle trafficking and secretory efficiency, whereas miRNA-375 inhibition facilitates effective insulin secretion [48]. Similarly, miRNA-375 targets Pdk1, a key kinase in the PI3K/Akt pathway that is essential for β-cell proliferation and survival. A deficiency in miRNA-375 has been linked to abnormal Pdk1 expression, leading to β-cell apoptosis and disrupted insulin signaling under metabolic and inflammatory stress [54]. Additionally, miRNA-375 targets Ras-related proteins, such as Rab27a, which are involved in the docking and exocytosis of insulin granules, further emphasizing its critical role in maintaining the integrity of the secretion machinery [11, 48].

Beyond the level of individual targets, miRNA-375 dynamically interacts with other islet-enriched miRNAs and pathways. Its synergistic actions with miRNAs, including miRNA-7, amplify β-cell-specific transcriptional networks, thereby enhancing insulin synthesis and secretion under normal physiological conditions [11, 48, 49]. Conversely, miRNA-375 antagonizes stress-associated miRNAs, such as miRNA-34a and miRNA-21, which are induced under inflammatory conditions and promote apoptosis and dysfunction. These antagonistic interactions form a protective mechanism that helps maintain β-cell viability. Furthermore, its involvement in the major signaling pathways PI3K/Akt and Mitogen-Activated Protein Kinase (MAPK) cascades parallels its role in regulating cellular growth, stress response, and survival [8, 50].

miR-375 does not operate in isolation; rather, its activity is intricately connected to a network of other islet miRNAs, often functioning in either synergistic or antagonistic manners. For example, miRNA-375 collaborates with miRNA-124 and miRNA-7 to regulate insulin biosynthesis and secretion. While miRNA-375 enhances insulin secretion by downregulating Mtpn, miRNA-124 complements this process by modulating vesicle trafficking proteins. Together, they optimize the exocytotic mechanism under normal physiological conditions. Conversely, miRNA-375 may exhibit antagonistic interactions with members of the miRNA-200 family, which are involved in β-cell stress responses and epithelial-mesenchymal transition (EMT)-related pathways. Under prolonged stress, the upregulation of miRNA-200 counteracts miRNA-375's role in maintaining β-cell identity, contributing to dedifferentiation and loss of function [55]. These interactions illustrate a complex network in which miRNA-375, along with other miRNAs, mediates β-cell function in response to metabolic and environmental signals.

Understanding these relationships could reveal potential therapeutic targets for interventions aimed at preserving β-cell functionality in diabetic patients. Importantly, dysregulation of miRNA-375 in T1DM contributes to a cascade of β-cell dysfunction, apoptosis, and increased vulnerability to autoimmune attack, underscoring its therapeutic potential. Targeting miRNA-375 and its associated network may offer novel strategies to restore β-cell function and slow the progression of T1DM [52].

## 6 THERAPEUTIC IMPLICATIONS OF MIRNA-375

The islet-enriched miRNA-375 is of great significance due to its role in β-cell function and its potential as a biomarker for T1DM [56-58]. In a study, miR-375 levels were quantified in 22 children with T1DM at disease onset and in 10 nondiabetic pediatric controls using a stem-loop reverse transcription polymerase chain reaction (RT-PCR) assay. The results indicated that serum miRNA-375 levels were significantly lower in patients than in controls, suggesting β-cell dysfunction. Furthermore, miRNA-375 levels were found to be independent of hemoglobin A1c (HbA1c), glycemia, or the presence of various autoantibodies, suggesting that dysregulation may not be influenced by immediate metabolic states or autoimmune factors [59].

In another study involving 66 patients with T1DM and 23 healthy controls, plasma levels of miRNA-375 were similarly decreased. This cohort was analyzed using the quantitative RT-qPCR method, revealing that miRNA-375 exhibited inverse correlations with levels obtained from HbA1c analyses. No significant relationships were found with disease duration, total daily insulin dosage, or specific autoantibodies, such as Glutamic Acid Decarboxylase Antibodies (GADA) and Islet Cell Antibodies (ICA) [60]. Another study compared the expression levels of miRNA-375 and miRNA-25 among T1DM patients, first-degree relatives (FDRs), and healthy controls. Notably, 35% of the T1DM patients showed poor expression of miRNA-375, while this reduced expression was also observed in 10% of the FDRs. Interestingly, although the mean miRNA-375 levels were lower in T1DM patients than in healthy controls, the FDRs showed higher levels. This finding may indicate an early stress state of the β-cells. Furthermore, miRNA-375 levels were found to strongly and inversely correlate with residual β-cell mass, positioning miRNA-375 as a potential marker of β-cell death and stress [61-63]. Additionally, miRNA-375 has been established as a marker for β-cell death in murine models. In streptozotocin-treated and NOD mice, circulating levels of miRNA-375 increased significantly before the development of hyperglycemia. Overall, miRNAs play a crucial role in the pathogenesis and potential diagnosis of T1DM [64].

A total of 90 patients with T1DM and 90 healthy matched controls were analyzed for the expression profiles of six plasma miRNAs, including miRNA-101-5p, miRNA-146-5p, miRNA-21-5p, miRNA-375, miRNA-126, and Let7a-5p. This analysis was conducted using RT-PCR and quantitative real-time PCR. A specific dysregulation pattern was identified. Notably, miRNA-101, miRNA-21, and miRNA-375 were significantly overexpressed in T1DM subjects compared to controls (*p* <0.05), indicating their involvement in β-cell stress, apoptosis, and immune dysregulation. Conversely, miRNA-146-5p, miRNA-126, and Let7a-5p were significantly downregulated, suggesting potential impairments in the resolution of islet inflammation, angiogenesis, and glucose metabolism. Further analysis revealed significant clinical correlations; miRNA-101 expression was directly correlated with age at diagnosis, potentially influencing the timing of disease onset. Additionally, miRNA-146-5p demonstrated a correlation with disease duration, suggesting its active involvement in chronic inflammatory processes associated with T1DM. Both miRNA-126 and Let7a-5p were identified through multiple regression analyses as robust biomarkers for predicting glycemic control parameters, with strong inverse relationships with HbA1c levels. The consensus in these findings reinforces the established roles of miRNA-126 in maintaining vascular integrity and Let7a-5p in regulating glucose homeostasis, thereby further validating their potential as biomarkers for disease progression and metabolic stability. These results highlight the utility of these six miRNAs as a biomarker panel for T1DM. The concurrent upregulation of miRNA-101, miRNA-21, and miRNA-375 emphasizes their potential as indicators of early β-cell damage and immune activation [65]. Conversely, the downregulation of miRNA-146-5p, miRNA-126, and Let7a-5p may indicate their involvement in the deterioration of long-term glycemic control and the complications associated with diabetes. These findings offer valuable insights not only into the pathophysiology of T1DM but also into enhancing diagnostic and monitoring techniques. Further studies are warranted to explore the combined predictive value of these miRNAs and their integration with established biomarkers, such as HbA1c, to refine clinical decision-making in T1DM management. The data strongly suggest that miRNA-375 serves as an early biomarker for β-cell apoptosis, occurring well before the clinical onset of diabetes [60-65].

However, miRNA-375 is not specific to β-cells. Dysregulation of miRNA-375 has also been implicated in viral infections, renal dysfunction, and α-cell proliferation in the pancreas, complicating its use as a standalone biomarker [66]. For instance, elevated levels of urinary miRNA-21, often detected alongside miR-375, are associated with an increased risk of kidney failure, indicating broader physiological implications beyond diabetes [67]. Collectively, these findings support the use of miRNA-375 as a biomarker for β-cell death and a predictor of diabetes, while also highlighting the need for multi-marker strategies to address its limited specificity [68-70].

Targeting miRNA-375 for therapeutic intervention in T1DM represents a promising approach to modulate disease progression and enhance β-cell preservation. miRNA-375 is highly expressed in pancreatic islets and plays a critical role in regulating β-cell function, proliferation, and apoptosis [71-73]. Dysregulation of miRNA-375 in T1DM has been consistently observed, with significantly lower levels in newly diagnosed patients compared to healthy individuals. Indeed, experiments have demonstrated that reduced miRNA-375 expression, associated with β-cell loss, suggests its involvement in the mechanisms underlying β-cell stress and cellular death. Therapeutic strategies targeting miRNA-375 could be driven by two main approaches: restoring its expression in β-cells or inhibiting its extracellular levels to mitigate inflammatory and apoptotic signaling [74]. In preclinical models, including streptozotocin-treated and NOD mice, elevated circulating levels of miRNA-375 preceded the onset of hyperglycemia and were closely associated with β-cell apoptosis. These findings indicate the potential to both prevent and treat through the modulation of miRNA levels. Restoring miRNA-375 expression in β-cells may enhance their regenerative capacity and improve insulin secretion. Conversely, reducing extracellular miRNA-375 levels, particularly during inflammatory episodes, may diminish its contribution to autoimmune processes [75-77].

The therapeutic potential of miRNA-375 is further emphasized by its involvement in various physiological pathways. It has been implicated in α-cell proliferation and the development of altered islet homeostasis following β-cell destruction, indicating a role in islet remodeling. Targeting miRNA-375 may be beneficial for restoring the balance of islet cell populations, thereby enhancing the functional capacity of residual β-cells while preventing compensatory α-cell proliferation [78].

## 7 CURRENT GAPS AND FUTURE RESEARCH DIRECTIONS

Several significant gaps exist in the current literature that require further investigation. First, no longitudinal studies have been reported to date that elucidate miRNA-375 levels in relation to β-cell destruction at various stages of disease progression. This research design would require recruiting individuals identified as high-risk for T1DM and monitoring them over an extended period. Additionally, it would require regular assessments of miRNA-375 levels in blood, serum, and other readily available biological fluids, conducted in parallel with the evaluation of β-cell function and mass through various methods, including C-peptide measurement, glucose tolerance tests, and advanced imaging techniques [54, 79]. Furthermore, it would be valuable to explore correlations between altered circulating miRNA-375 levels and markers of β-cell destruction and autoimmune activity. Importantly, it will be essential to identify critical time points or thresholds in miRNA-375 expression that precede accelerated β-cell loss and/or disease progression, as well as to further investigate the potential role of miRNA-375 as a predictive biomarker for the onset and prognosis of T1DM [80-82].

Future research should explore how genetic susceptibility and environmental risk factors interact with miRNA-375 in the pathogenesis of T1DM [83, 84]. This may involve elucidating miRNA-375 expression in genes associated with T1DM susceptibility. Additionally, future studies should examine the impact of external factors, such as viral infections, dietary habits, and stress, on miRNA-375 levels and function in β-cells [85]. The mechanisms by which environmental exposures alter miRNA-375 expression through epigenetic modifications, as well as how miRNA-375 further influences the function of immune cells and autoimmune responses, remain poorly understood. Animal models that incorporate both genetic and environmental factors, including miRNA-375 expression, may provide a more comprehensive understanding of T1DM in humans. Further investigation of miRNA-375 as a link between genetic and environmental factors in T1DM could be highly significant [86].

The development of reliable and non-invasive methods for monitoring miRNA-375 levels is crucial for translating research findings into practical clinical tools. Future studies should focus on developing highly sensitive and specific assays to measure miRNA-375 in readily accessible fluids, such as blood, urine, or saliva [87]. Liquid biopsy techniques that analyze circulating exosomes or other vesicles for miRNA-375 may be particularly significant. Additionally, the relationship between miRNA-375 levels in various fluids and pancreatic tissue must be examined from multiple perspectives to identify optimal sampling methods [88, 89]. Designing diverse instruments suitable for point-of-care testing (POCT) or rapid assays that incorporate clinical measurements of miRNA-375 will greatly enhance diagnostic possibilities. Advanced molecular imaging techniques using probes may enable intriguing, non-invasive observations of miRNA-375 expression levels in the pancreas. Furthermore, the development of algorithms and computational tools to analyze fluctuations in miRNA-375 levels and their impact on β-cell function will be essential for advancing both understanding and clinical applications [44, 45]. More specifically, in the T1DM model, a series of more recent reports substantiates an additional prognostic (or diagnostic at an early time point) role for miR-375. It was found that children with T1D and, indeed, first-degree relatives of children with T1D have significantly reduced circulating miR-375 compared to healthy controls, that circulating miR-375 correlates negatively with HbA1c, and that, inferentially, reduced miR-375 relates to worse glycaemic control or more β-cell dysfunction [9]. Besides, children with T1D diagnosed recently have lower miR-375 levels than controls, suggesting low miR-375 at short disease duration [60]. Long-term follow-up investigations must establish whether sustained miR-375 levels in risk groups (*e.g*., autoantibody-positive individuals or sibs) are a consistent predictor of disease onset or β-cell loss. Standard measurements and large prospective cohorts should also be used to assess the prognostic precision (sensitivity/ specificity) of miR-375 and to establish its relative merit compared with other existing risk markers.

## CONCLUSION

miRNA-375 plays a crucial role in β-cell function and the pathogenesis of autoimmune destruction in T1DM. It regulates insulin secretion, β-cell survival, and glucose homeostasis by targeting key genes, including Mtpn, Pdk1, and Ras-related proteins. Additionally, its interactions with other microRNAs and signaling pathways, including PI3K/Akt and MAPK, further emphasize its central role in maintaining β-cell integrity. Dysregulation of miRNA-375 contributes to β-cell dysfunction and increases susceptibility to immune-mediated damage in T1DM. These findings highlight miRNA-375 as a potential biomarker and therapeutic target for preserving β-cell function and mitigating the development of T1DM.

## Author’s Contributions

The authors confirm contribution to the paper as follows: study conception and design: SK, RFS, HIT, and TMA; data collection: SK, RFS, HIT, TMA, and JZA; analysis and interpretation of results: SK, HIS, TA, RFS, HIT, TMA, and MEM; draft manuscript: SK, HIS, TA, RFS, HIT, TMA, JZA, and MEM. All authors reviewed the results and approved the final version of the manuscript.

## Figures and Tables

**Fig. (2):**
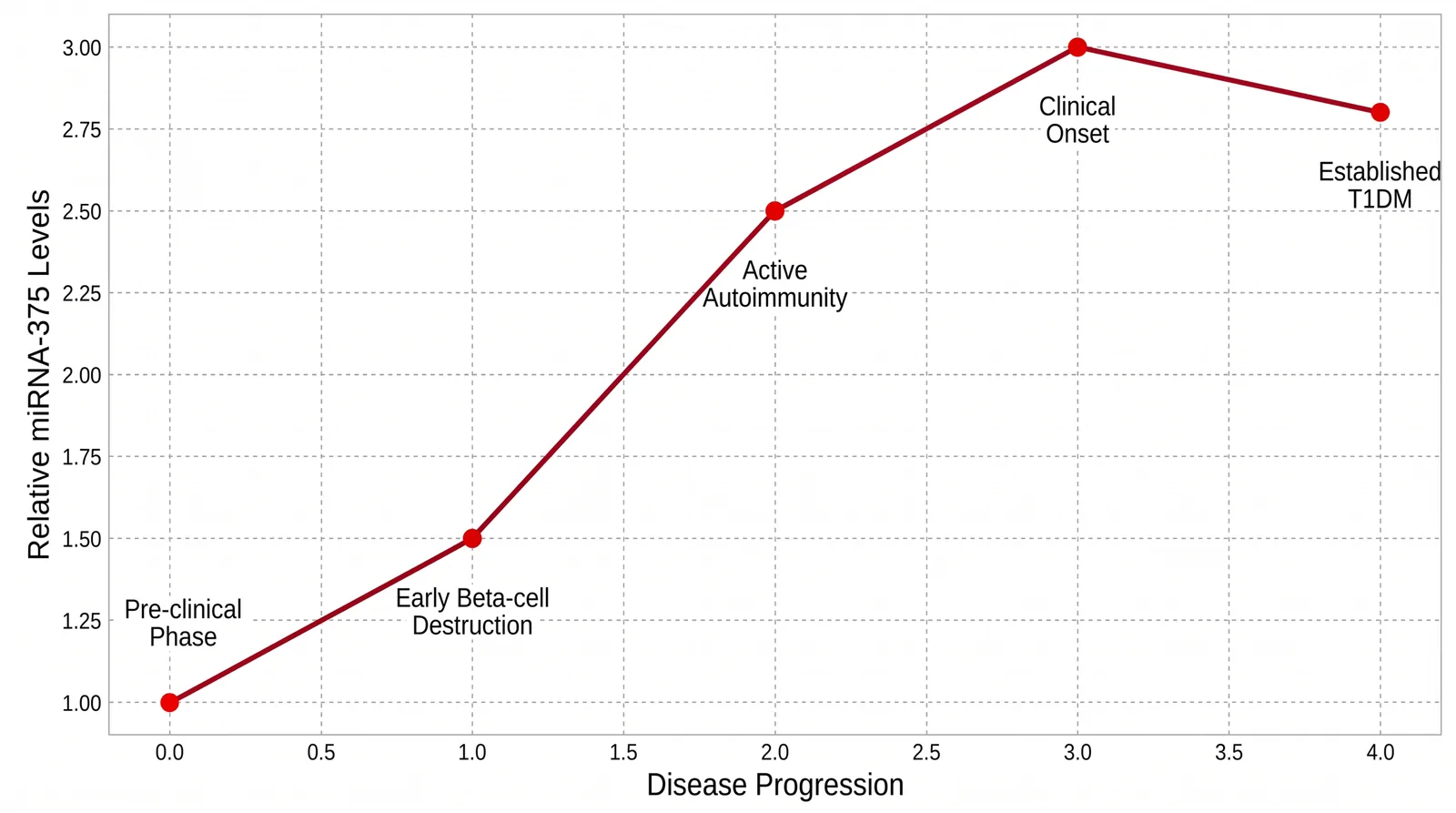
Changes in circulating miRNA-375 levels during T1DM progression [43-45].

## LIST OF ABBREVIATIONS

3′-UTRs: 3′-Untranslated Regions
Bim: Bcl-2-interacting Mediator of Cell Death
EMT: Epithelial-mesenchymal Transition
FDRs: First-degree Relatives
GADA: Glutamic Acid Decarboxylase Antibodies
HbA1c: Hemoglobin A1c
ICA: Islet Cell Antibodies
IFN-γ: Interferon Gamma
IL-1β: Interleukin 1 Beta
KO: Knockout
MAPK: Mitogen-Activated Protein Kinase
miRNAs: microRNAs
mRNA: Messenger RNA
NF-κB: Nuclear Factor Kappa B
NOD: Non-obese Diabetic
Pdk1: Phosphoinositide-dependent Protein kinase-1
POCT: Point-of-care Testing
pre-miRNA: Precursor miRNA
RISC: RNA-induced Silencing Complex
RT-PCR: Reverse Transcription Polymerase Chain Reaction
STAT3: Signal Transducer and Activator of Transcription 3
T1DM: Type 1 Diabetes Mellitus
T2DM: Type 2 Diabetes Mellitus
TNF-α: Tumor Necrosis Factor Alpha
Tregs: Regulatory T Cells
YAP1: Yes-associated Protein 1

## DISCLAIMER

Please note that this article is currently available in the Early View stage but represents the final Version of Record (VoR). This is the definitive, fully citable version, which has undergone copyediting, typesetting, metadata assignment, and DOI allocation. The Version of Record (VoR) is considered final and is not subject to further content changes, except for the assignment of volume, issue, and page numbers when it is formally incorporated into a specific journal issue. Despite this, the article already carries complete bibliographic details and can be cited confidently prior to its formal placement in an issue.
